# Supplementary material for: PDZK1-Interacting Protein 1(PDZKIP1) Inhibits Goat Subcutaneous Preadipocyte Differentiation through Promoting Autophagy
Source: Animals (Basel). 2023 Mar 14;13(6):1046. doi: 10.3390/ani13061046 (PMC10044287; doi:10.3390/ani13061046)

Figure S1. Western Original Image

Full original blots used for Figure 4A. Each blot membrane was cut based on the standard band positions and then incubated with the appropriate antibodies.
The figure in the article was marked with red box and the corresponding bands were marked by red lines.

Figure 4A

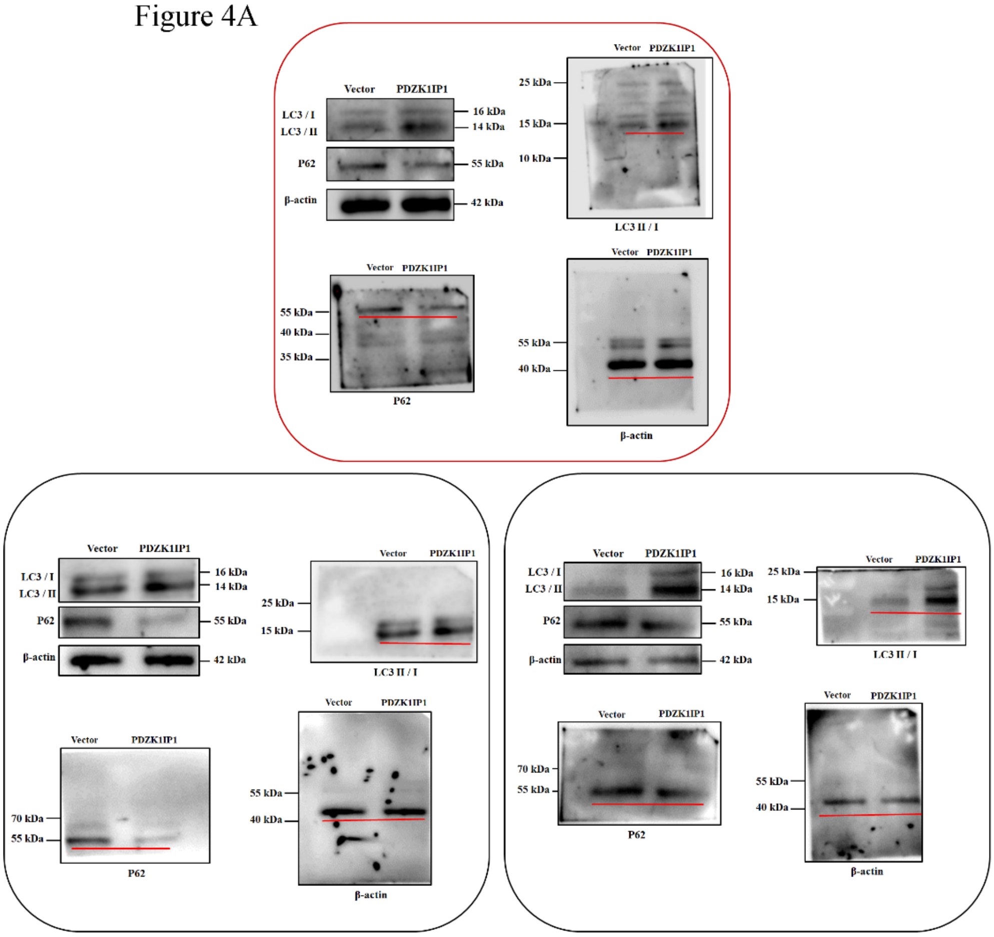

Full original blots used for Figure 5A. Each blot membrane was cut based on the standard band positions and then incubated with the appropriate antibodies.
The figure in the article was marked with red box and the corresponding bands were marked by red lines.

Figure 5A

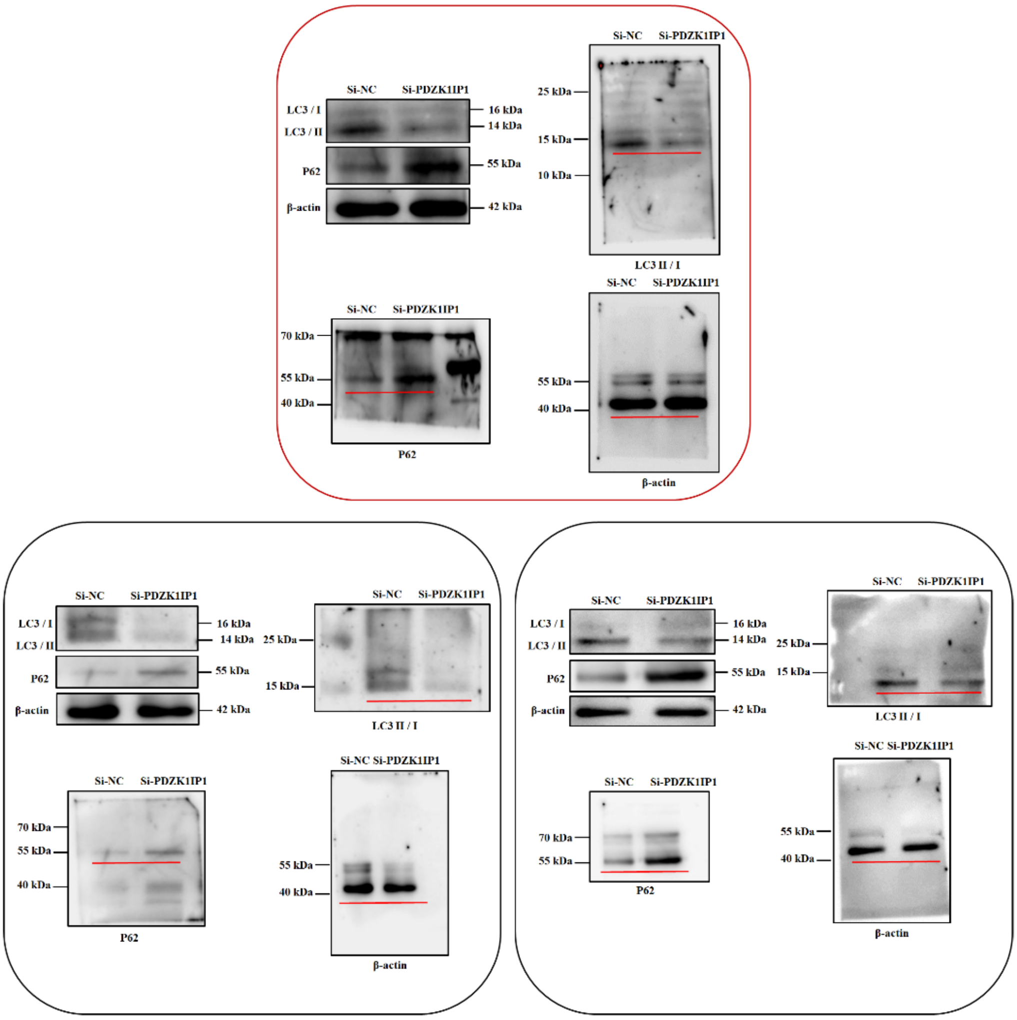

Full original blots used for Figure 6A. Each blot membrane was cut based on the standard band positions and then incubated with the appropriate antibodies.

The figure in the article was marked with red box and the corresponding bands were marked by red lines.

Figure 6A

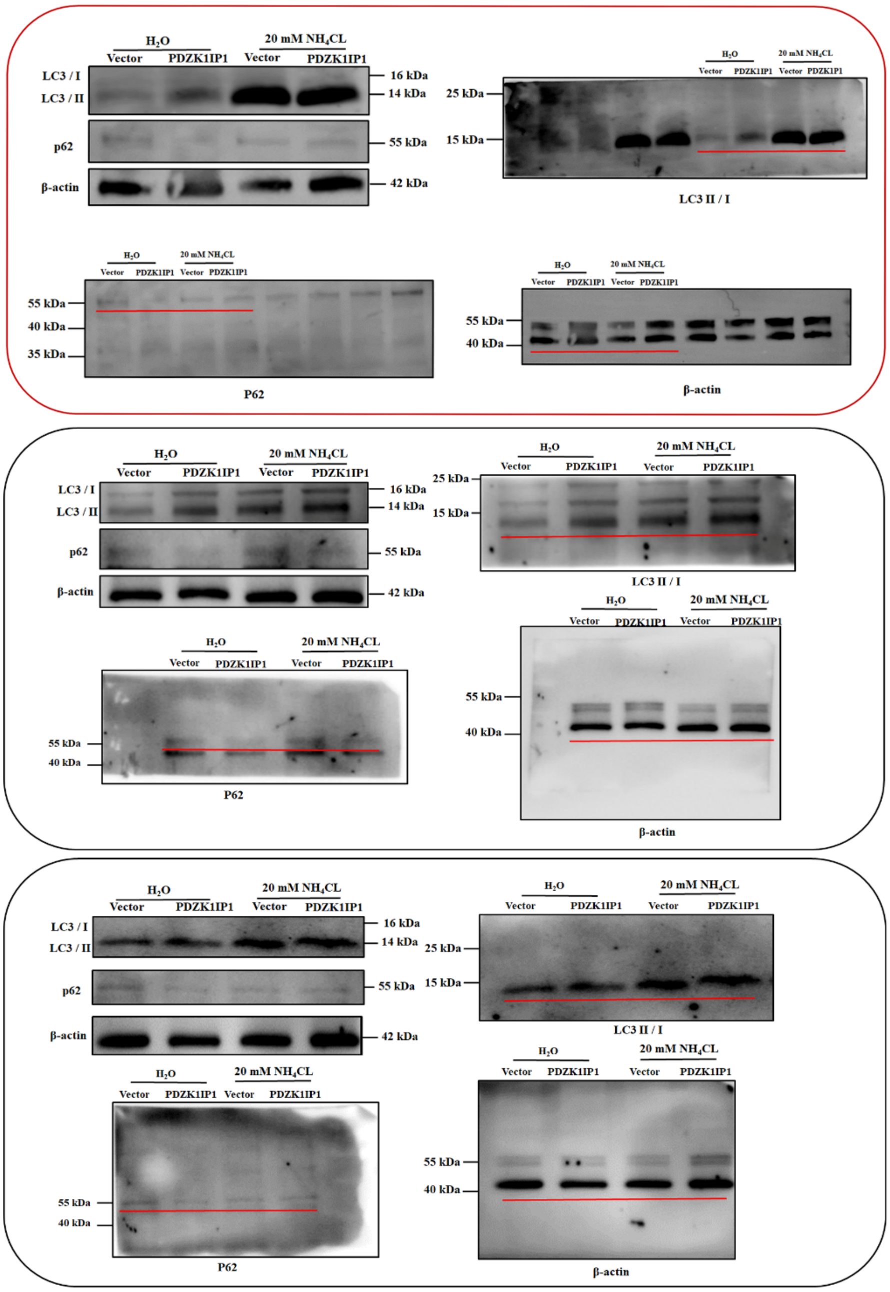

Supplement: Supplementary file 1 [file animals-13-01046-s001.zip › animals-2241653-supplementary.pdf]
